# Butein promotes ubiquitination-mediated survivin degradation inhibits tumor growth and overcomes chemoresistance

**DOI:** 10.1038/s41598-022-21839-4

**Published:** 2022-11-30

**Authors:** Xin Dong, Wenbin Liu, Xiaoying Li, Yu Gan, Li Zhou, Wei Li, Li Xie

**Affiliations:** 1 Department of Head and Neck Surgery, Hunan Cancer Hospital/The Affiliated Cancer Hospital of Xiangya School of Medicine, Central South University, Changsha, 410013 Hunan China; 2 Department of Pathology, Hunan Cancer Hospital/The Affiliated Cancer Hospital of Xiangya School of Medicine, Central South University, Changsha, 410013 Hunan China; 3 Department of Pathology, National Clinical Research Center for Geriatric Disorders, Xiangya Hospital of Central South University, Changsha, 410008 Hunan China; 4 Cell Transplantation and Gene Therapy Institute, The Third Xiangya Hospital, Central South University, Changsha, 410013 Hunan China; 5 Department of Clinical Laboratory, National Cancer Center/National Clinical Research Center for Cancer/Cancer Hospital, Chinese Academy of Medical Sciences and Peking Union Medical College, Beijing, 100021 China

**Keywords:** Cancer, Oncology

## Abstract

Overexpression of survivin is frequently observed in human malignancies and is associated with poor prognosis. The present study found that survivin is highly expressed in nasopharyngeal carcinoma (NPC) tumor tissues. Depleting survivin with shRNA inhibited cell viability, colony formation, and in vivo tumorigenesis of NPC cells. With a natural product screening, we identified Butein as a potential anti-tumor compound for NPC by reducing survivin protein level. Butein shortened the half-life of survivin and enhanced ubiquitination-mediated degradation. The mechanism study showed that Butein promoted the interaction between survivin and E3 ligase Fbxl7, and the knockdown of Fbxl7 compromised Butein-induced survivin ubiquitination. Butein suppressed the Akt-Wee1-CDK1 signaling and decreased survivin Thr34 phosphorylation, facilitating E3 ligase Fbxl7-mediated survivin ubiquitination and degradation. Moreover, Butein exhibited a strong in vivo anti-tumor activity, as the tumor volume of Butein-treated xenografts was reduced significantly. Butein alone or combined with cisplatin (CDDP) overcame chemoresistance in NPC xenograft tumors. Overall, our data indicate that Butein is a promising anti-tumor agent for NPC treatment.

## Introduction

Nasopharyngeal carcinoma (NPC) is one of the most commonly diagnosed malignant tumors that arise from the nasopharynx's epithelial lining. The incidence of NPC exhibits prominent ethnic, regional, and familial epidemiological characteristics. NPC causes a heavy public-health burden in Southeast Asia, Southern China, the Arctic, North Africa, and the Middle East, and approximately 47.7% of all new cases of NPC globally were diagnosed in Southern China^1,2^. To date, the precise mechanism of NPC tumorigenesis remains unclear. However, a panel of risk factors has been described, including ethnicity, EBV infection, hereditary trends, dietary habits, and tobacco consumption^2–4^. Due to NPC's special location and occult nature, over 70% of patients were diagnosed at an advanced stage. Although concurrent radiationtherapy/chemotherapy significantly improves the prognosis of NPC, metastasis is the leading cause of therapy failure and cancer-related death^2,5–7^. Thus, further elucidating the mechanisms of NPC tumorigenesis and discovery of novel anti-tumor targets are still the urgent demand for NPC treatment.

Survivin plays a crucial role in G2/M cell cycle transition and apoptosis inhibition^8^. Survivin binds with Aurora B, INCENP, and Borealin to form the chromosomal passenger complex (CPC), which guarantees proper chromosomes and segregation during mitosis^9^. Accumulating evidence indicates that survivin is frequently overexpressed in human cancers, including non-small cell lung cancer^10^, prostate cancer^11^, colorectal cancer^12^, gastric cancer^13^, and hepatocellular carcinoma^14^. Highly expressed survivin is associated with radio/chemotherapy resistance, tumor recurrence, and poor prognosis, making survivin a fantastic therapeutic target for anticancer treatment^15^. Survivin localizes in both cytoplasm and nuclear and exhibits various subcellular functions. Survivin is predominantly expressed in the cytoplasm and binds with caspase-3 to maintain the survival of cancer cells, whereas the nuclear-localized survivin is an essential subunit of CPC complex to orchestrate mitosis^16,17^. Thus, as an oncoprotein that is both required for cell cycle progression and apoptosis suppression, survivin is emerging as a promising anti-tumor target for clinical treatment.

The natural product Butein is a potent anti-tumor agent against human malignancies^17,18^. Previous studies have shown that Butein suppresses osteosarcoma^19^, cervical cancer^20,21^, colorectal cancer^22^, hepatocellular carcinoma^23^, breast cancer^24,25^, lung cancer^26^, and oral squamous cell carcinoma^27^. The mechanism studies have identified multiple intracellular targets of Butein, including AurkB, mTOR, NF-κB, EGFR, and c-Met^17,28–30^. Butein induces cell cycle arrest, promotes apoptosis, suppresses angiogenesis/metastasis, and compromises glycolysis^17^. However, the antitumor effect of Butein on NPC and the potential synergistic function of Butein on chemoresistance is unclear.

In this study, we found that survivin is overexpressed in NPC tissues and required for maintaining the malignant phenotype of NPC cells. We demonstrated that the natural product Butein is a potential anti-tumor compound for NPC treatment. We investigated the inhibitory effect of Butein on NPC cells and determined the underlying mechanism of this anti-tumor efficacy.

## Materials and methods

### Cell culture and antibodies

The chemicals, including the natural product Butein, cycloheximide (CHX), and proteasome inhibitor MG132, were obtained from Selleck Chemicals (Houston, TX). Human NPC cell lines, including CNE2 (a low-differentiated NPC epithelial cell line derived from a primary tumor biopsy), HONE1 (latently infected with Epstein-Barr virus and derived from primary nasopharyngeal carcinomas), and SUNE1 (epithelial cancer cells derived from primary nasopharyngeal carcinomas), were purchased from the Cell Bank of Central South University, Changsha, China, and maintained in a 37 °C humidified incubator with 5% CO_2_ according to the standard protocols. All cell lines were routinely tested for mycoplasma contamination and subjected to a cytogenetic test to confirm identity. Cell culture medium and supplies, including RPMI-1640 and Fetal Bovine Serum (FBS), were obtained from Invitrogen (Grand Island, NY). The primary antibodies against survivin (#2808), cleaved-caspase 3 (#9664), cleaved-PARP (#5625), cytochrome C (#11940), Bax (#5023), VDAC1(#4661), α-Tubulin(#3873), ubiquitin (#3936), Flag-tag (#8146), HA-tag (#3724), and β-actin (#4970) were purchased from Cell Signaling Technology, Inc. (Beverly, MA). The anti-ki67 (ab16667) and FbxL7 (#ab59149) antibodies were products of Abcam (Cambridge, United Kingdom). The Flag-Survivin construct was purchased from Origene (RC205935), and the T34A mutant was generated using the Q5 Site-Directed Mutagenesis kit (Cat. #E0554S, NEB, Ipswich, MA) following the manufacturer’s standard protocol and verified by DNA sequencing.

### Cell viability assay

The MTS assay was performed to analyze cell viability. Briefly, NPC cells were counted and seeded into the 96-well plates at 3000 cells/well density and maintained overnight. Cells were exposed to different doses of Butein and culture for 24, 48, and 72 h. The MTS reagent (#G3580, Madison, WI) was added to the cell culture medium, and cell viability was examined following the standard protocol.

### Anchorage-independent cell growth

The colony formation in soft agar was performed as described previously^18^. Briefly, the 0.6% agar containing Eagle’s basal medium was mixed with 10% FBS, and various dose of Butein was used as an agar base in a 6-well plate. The cells were counted and resuspended at a density of 8000 cells/ml using the Eagle’s basal medium containing 0.3% agar, 10% FBS, and various concentration of Butein. The mixture was overlaid into a 6-well plate with a 0.6% agar base and maintained for 2 weeks for colony counting.

### Clinical tissue sample collections

A total of 30 cases of NPC tissues and matched adjacent non-tumor tissues were collected from 30 patients with written informed consent by the Department of Pathology, Hunan Cancer Hospital of Central South University, Changsha, Hunan, China. All the patients received no treatment before surgery.

### Western blotting

The cells were treated with Butein, and the whole-cell extract (WCE) was prepared using the commercial RIPA buffer (#89901, Thermo Fisher Scientific) with proteasome inhibitor. WCE was concentrated using the BCA protein assay kit (#23228, Thermo Fisher Scientific) and subjected to immunoblotting (IB) analysis. Briefly, WCE (a total of 15 μg) was boiled with loading buffer at 95 °C for 5 min and subjected to SDS-PAGE electrophoresis and electrotransfer to the PVDF membrane. After being blocked with 5% non-fat milk, the membrane was incubated with primary and second antibodies. The ECL substrate (#34579, Thermo Fisher Scientific) was used for target protein visualization. The membranes were cut prior to hybridization with antibodies, therefor the images of adequate length were absence. All uncropped western blots results were included in Supplemental Fig. 4.

### Natural compound screening

The 82 compounds of interest were selected from the Natural Product Library (Cat. No. L1400-01/02) from Selleck Chemicals (Houston, TX). CNE2 cells were seeded in a 96-well plate. After overnight incubation, cells were treated with DMSO (control) or natural compounds (5 µM) for 48 h. Cell viability was examined by the MTS assay. Tested compounds are listed in Supplementary Table 1.

### Generation of survivin knockdown stable cell lines

To generate survivin knockdown stable cells, *pLKO.1-shsurvivin* lentivirus plasmids (TRCN0000073718, TRCN0000073721, Millipore Sigma) were cotransfected into 293 T cells with *PSPAX2* and *PMD2-G*. Three days later, the viral supernatant fractions were harvested and filtered through a 0.45 mm filter, followed by infection into cells with 5 mg/mL polybrene. The puromycin (1 μg/ml) containing fresh medium was replaced 36 h after infection and maintained for 1 week for colony selection.

### In vivo tumor growth

The xenograft mouse model was approved by the Institutional Animal Care and Use Committee (IACUC) of Central South University (Changsha, China). In vivo tumor growth was performed by s.c.injection of CNE2 cells (3 × 10^6^) into the right flank of 7-week-old athymic nude mice (n = 5). Tumor volume was recorded, and Butein administration was initiated when tumor volume reached 100 mm^3^. The control group was administered the vehicle control, whereas the compound-treated group was administered Butein (20 mg/kg) every 2 days by i.p. injection. For the combination treatment, the tumor-bearing mice were randomly divided into four groups (n = 5): 1,vehicle control (0.5% dimethyl sulfoxide, 100 µL/every 2 days, i.p.); 2, Butein (10 mg/kg/ every 2 days, i.p.); 3, CDDP (3 mg/kg/ every 2 days, i.p.); 4, Butein (10 mg/kg/ every 2 days, i.p.) + CDDP (3 mg/kg/ every 2 days, i.p.). The mice were euthanized with CO_2_ (3 L/min) for 5 min at the endpoint. Tumor volume was determined using the following formula: length × width × width/2.

### Immunohistochemical staining (IHC)

Tumor tissues from clinical samples and mouse xenograft tumors were fixed and subjected to IHC analysis as described previously^19^. Briefly, the tissue slides were deparaffinized and rehydrated by incubation with xylene and ethanol, then submerged into sodium citrate buffer (10 mM, pH 6.0) and boiled for 10 min for antigen retrieval. The activity of endogenous horseradish peroxidase was quenched by incubation with 3% H_2_O_2_ in methanol for 10 min. Tissue slides were washed with PBS and blocked with 50% goat serum albumin. After incubating the primary and second antibodies, the target protein was visualized using the DAB substrate and counterstained by hematoxylin.

### Statistical analysis

Statistical analysis was performed using GraphPad Prism 5 (GraphPad 5.0, San Diego, CA, USA). All quantitative data were performed from three independent experiments and expressed as mean ± sd. The difference was evaluated using the Student's t-test or ANOVA. A probability value of p < 0.05 was used as the criterion for statistical significance.

### Ethics approval and consent to participate

The animal experiments were approved by the Medical Research Animal Ethics Committee, Central South University, China.

## Results

### Survivin is highly expressed in nasopharyngeal carcinoma and is required for maintaining tumorigenic properties

**Figure 1:**
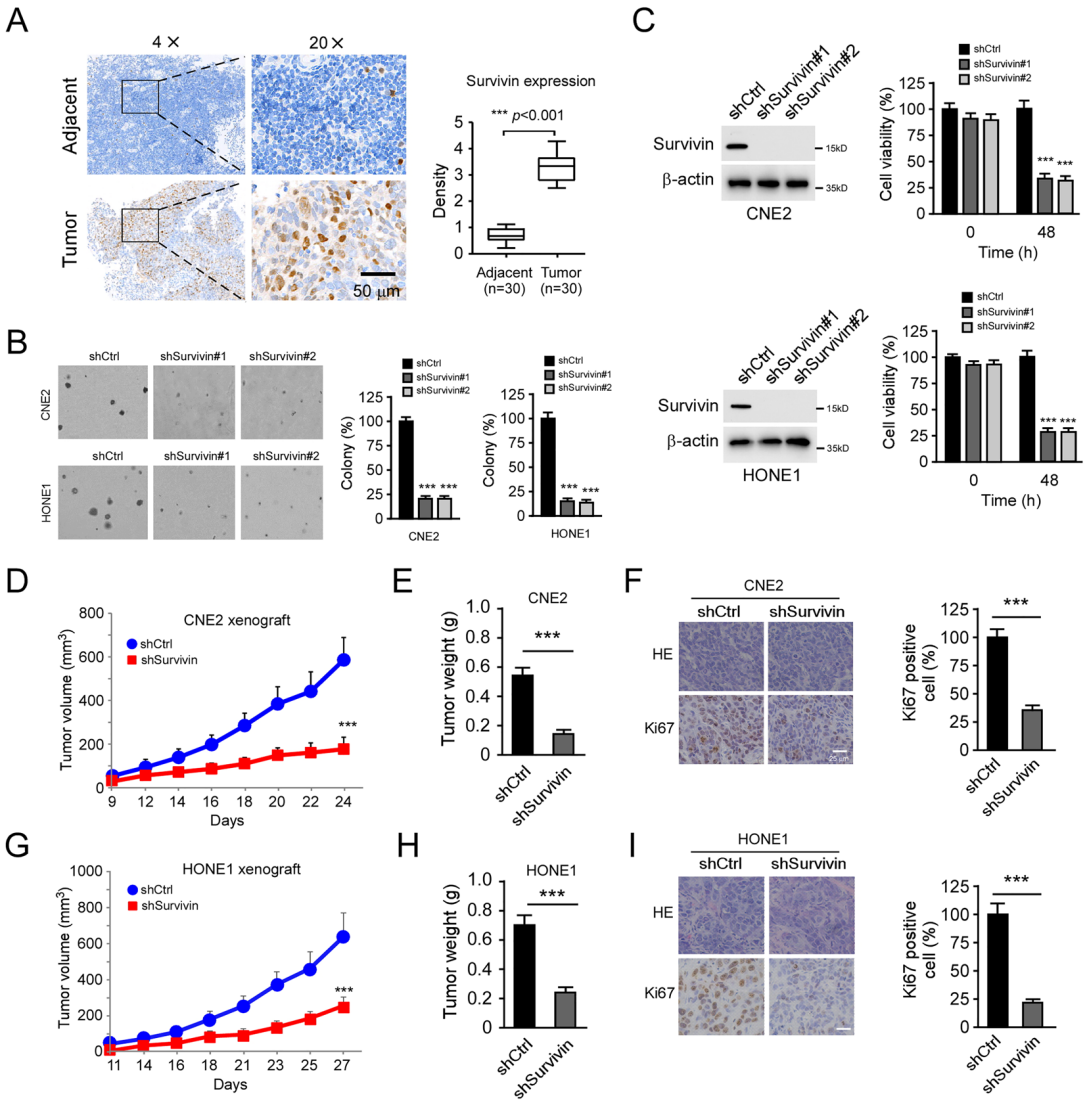
Survivin is required for the malignant phenotype of NPC cells. (**A**) IHC staining analysis of survivin expression in 30 cases of matched NPC patient tissues and adjacent non-tumor tissues. Left, the representative IHC staining results of survivin. Right, Qualification analysis of survivin expression. Scale bar, 50 μm. (**B**) The colony formation of CNE2 and HONE1 cells expressing shCtrl or shsurvivin. (**C**) Cell viability of CNE2 (top) and HONE1 (bottom) cells expressing shCtrl or shsurvivin. Left, immunoblotting (IB) analysis of survivin protein level. Right, MTS analysis of cell viability. (**D**–**F**) In vivo tumorigenesis of CNE2 expressing shCtrl or shsurvivin. Tumor volume (**D**), tumor weight (**E**), and IHC staining analysis of the population of Ki67 positive cells (**F**) of the CNE2 xenografts. Scale bar, 25 μm. ***p < 0.001. (**G**–**I**) In vivo tumorigenesis of HONE1 expressing shCtrl or shsurvivin. Tumor volume (**G**), tumor weight (**H**), and IHC staining analysis of the population of Ki67 positive cells (**I**) of the HONE1 xenografts. Scale bar, 25 μm. ***p < 0.001.

To investigate the oncogenic function of survivin in nasopharyngeal carcinoma (NPC), we determined the protein level of survivin in tumor tissues. The result showed that survivin is overexpressed in NPC tissues compared to the paired non-tumor adjacent tissue (Fig. 1A). We next constructed survivin knockdown stable cells in CNE2 and HONE1 cells. The colony formation assay revealed that the depletion of survivin inhibited anchorage-independent cell growth of CNE2 and HONE1 cells, as the colony number was reduced by around 70% compared to that of survivin proficient cells (Fig. 1B). Consistently, the MTS data indicated that knockdown of survivin significantly suppressed the cell viability of NPC cells (Fig. 1C). We next examined the in vivo tumorigenesis of survivin knockdown cells. The xenograft model showed that depletion of survivin inhibited tumor development. The CNE2-shsurvivin-derived xenograft tumors exhibited smaller tumor volume and weight (Fig. 1D–F). The IHC data showed that the population of Ki67 positive cells was attenuated significantly in shsurvivin-expression xenograft tumors (Fig. 1G). Our results suggest that survivin is highly expressed in NPC tissues, and knockdown of survivin impaired the tumorigenic properties of NPC cells.

### Butein inhibits NPC cells in vitro

**Figure 2:**
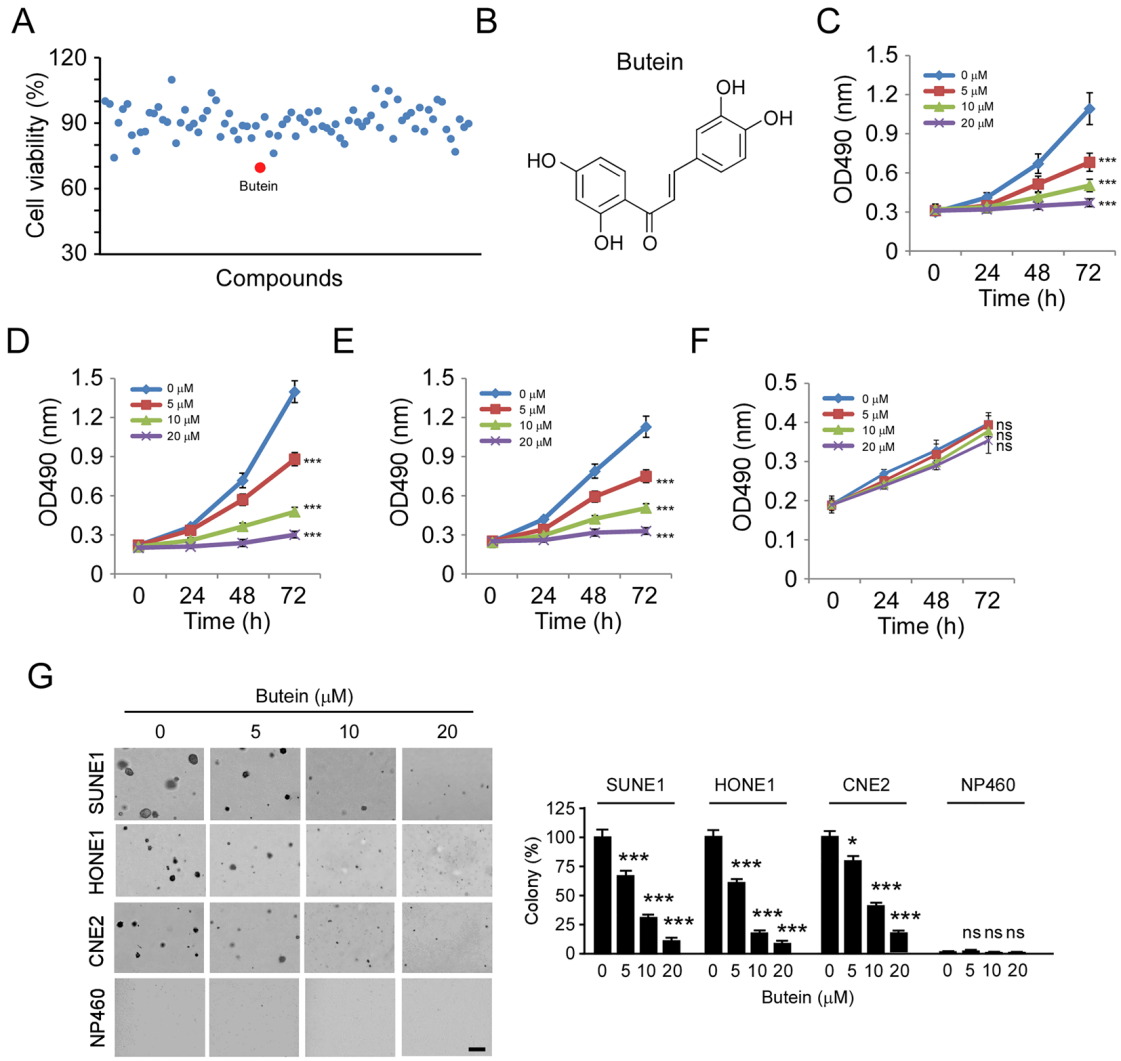
Butein inhibits NPC cells in vitro. (**A**) CNE2 cells were treated with the screened compounds for 48 h. Cell viability was examined by MTS assay. Red dot, Buttein. (**B**) the chemical structure of Butein. (**C**–**E**) MTS assay analysis of the cell viability of CNE2 (**C**), SUNE1 (**D**), and HONE1 (**E**) cells with Butein treatment. (**F**) MTS assay analysis of the cell viability of NP460. (**G**) Soft agar assay analysis of the colony formation of CNE2, SUNE1, HONE1, and NP460 cells. *p < 0.05, ***p < 0.001. ns, not statistically significant.

To discover natural compounds (Supplementary Table 1) that can suppress NPC cells and reduce survivin protein levels, we screened a customized natural compound library containing 82 compounds of interest by MTS assay. We found that only Butein caused a reduction of cell viability in CNE2 cells by around 30% at a single dose of 5 μM (Fig. 2A, Supplementary Fig. 1). Butein (Fig. 2B) exhibits strong anti-tumor activities in human cancer models. However, the anti-tumor potential of Butein on NPC cells is not clear. We found that Butein significantly inhibited the cell viability of CNE2, SUNE1, and HONE1 cells dose-dependently (Fig. 2C–E). Exposure to Butein (20 μM) for 72 h decreased the cell viability of all tested NPC cells by over 80%. However, Butein failed to inhibit the cell viability of immortalized non-tumor cell NP460 (Fig. 2F). We next tested the antitumor activity of Butein using the soft agar assay. The non-tumor cell NP460 failed to form the colony in the soft agar (Fig. 2G). However, the efficacy of colony formation of NPC cells in soft agar was impaired significantly after Butein treatment. The inhibitory effect of Butein was enhanced dose-dependently, and 10 μM Butein reduced the colony number by over 60% in CNE2, HONE1, and CNE1 cells. Moreover, 20 μM Butein decreased the colony number by over 85% in SUNE1 and HONE1 cells and over 75% in CNE2 cells, respectively (Fig. 2G). These data indicate that Butein inhibits NPC cells in vitro.

### Butein promotes survivin degradation in NPC cells

**Figure 3:**
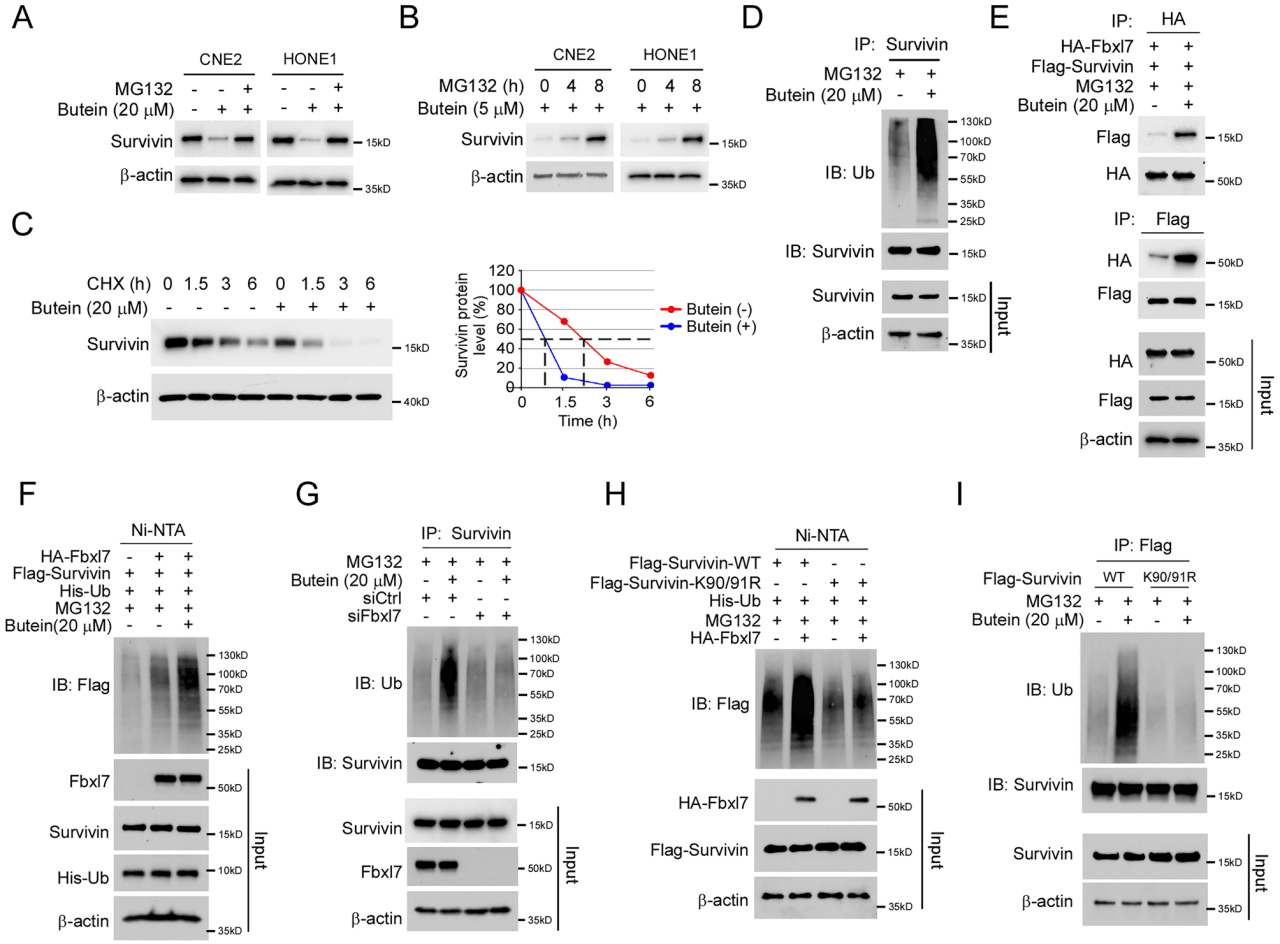
Butein promotes survivin ubiquitination. (**A**) CNE2 and HONE1 cells were treated with Butein for 24 h, and incubated with MG132 (20 µM) for 8 h. The whole-cell extract (WCE) was subjected to IB analysis. (**B**) CNE2 and HONE1 cells were treated with Butein for 24 h, and incubated with MG132 (20 µM) for various time points. The WCE was subjected to IB analysis. (**C**) CNE2 cells were treated with Butein for 24 h, and incubated with CHX for different time points. The WCE was subjected to IB analysis. (**D**) CNE2 cells were treated with Butein for 24 h, and incubated with MG132 (20 µM) for 8 h. The WCE was subjected to survivin ubiquitination analysis. (**E** and **F**) CNE2 cells were transfected with various constructs for 24 h, followed by Butein treated for 24 h, and incubated with MG132 (20 µM) for 8 h. The WCE was subjected to co-IP (**E**) or survivin ubiquitination (**F**) analysis. (**G**) CNE2 cells were transfected with siFbxl7 for 24 h and treated with Butein for another 24 h. WCE was collected after the cells were incubated with MG132 (20 µM) for 8 h. The WCE was subjected to survivin ubiquitination analysis. (**H** and **I**) CNE2 cells were transfected with various constructs for 24 h, followed by Butein treated for 24 h, and incubated with MG132 (20 µM) for 8 h. The WCE was subjected to survivin ubiquitination analysis.

We next determined the mechanisms of how Butein inhibited NPC cells in vitro. The Immunoblotting data showed that treatment of MG132, a proteasome inhibitor, restored the protein level of survivin in Butein-treated NPC cells (Fig. 3A,B), indicating that Butein might affect the stability of survivin protein. As shown in Fig. 3C, exposure to Butein reduced the half-life of survivin in CNE2 cells. We next determined the ubiquitination of survivin in Butein-treated cells. The result showed that Butein promoted survivin ubiquitination in CNE2 and HONE1 cells (Fig. 3D, Supplementary Fig. 2A). Furthermore, the co-IP data indicated that treatment with Butein enhanced the interaction between survivin and the E3 ligase Fbxl7 (Fig. 3E). The ubiquitination assay revealed that Butein enhanced Fbxl7-mediated survivin ubiquitination (Fig. 3F, Supplementary Fig. 2B). To determine whether Fbxl7 is required for survivin ubiquitination in Butein-treated NPC cells, we silenced Fbxl7 using siRNA in CNE2 cells. We found that knockdown of Fbxl7 impaired Butein-induced survivin ubiquitination (Fig. 3G, Supplementary Fig. 2C). Mutation of the residues K90/91 compromised Fbxl7-induced survivin ubiquitination in CNE2 and HONE1 cells (Fig. 3H, Supplementary Fig. 2D), which is consistent with previous studies that K90/91 plays crucial role in survivin stability. Furthermore, our data showed that mutation of K90/91 reduced Butein-induced survivin ubiquitination in CNE2 and HONE1 cells (Fig. 3I, Supplementary Fig. 2E). These results imply that Butein promotes survivin ubiquitination and degradation.

### Butein-decreased Akt phosphorylation is required for survivin ubiquitination

**Figure 4:**
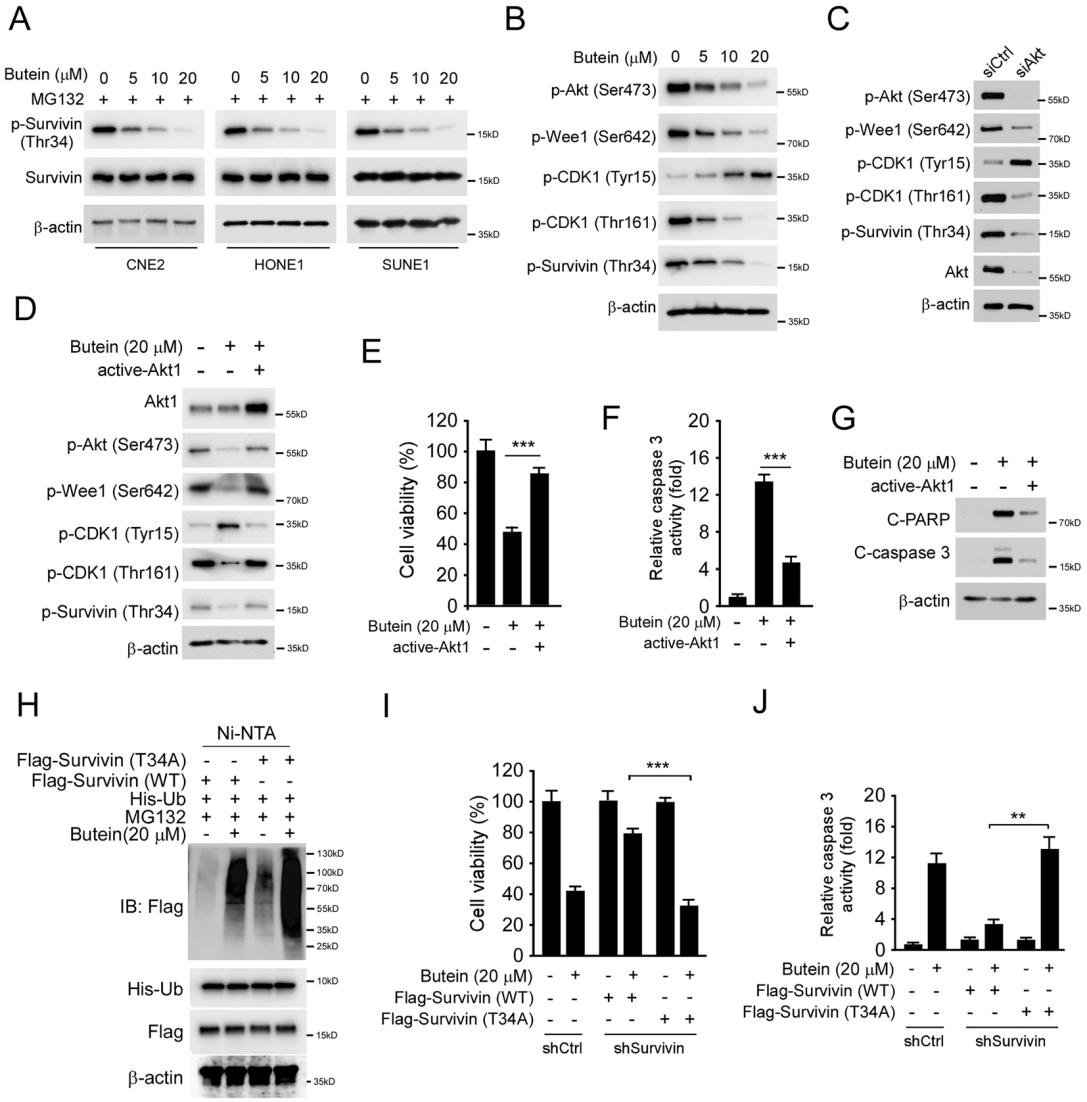
Butein suppressed Akt activity and reduced survivin Thr34 phosphorylation. (**A**) CNE2, SUNE1, and HONE1 cells were treated with Butein for 24 h, followed by MG132 treated for 8 h, and the WCE was subjected to IB analysis. (**B**) CNE2 cells were treated with Butein for 24 h. The WCE was subjected to IB analysis. (**C**) CNE2 cells were transfected with siAkt for 48 h. The WCE was subjected to IB analysis. (**D**–**G**) CNE2 cells were transfected with constitutively activated Akt1 for 24 h, followed by Butein treated for another 24 h, the WCE was subjected to IB analysis (**D** and **G**), cell viability was determined by MTS assay (**E**), and caspase 3 activity was examined by Caspase 3 Assay Kit (**F**). ***p < 0.001. H, CNE2 cells were transfected with various constructs for 24 h and treated with Butein for another 24 h. MG132 was added to the cell culture medium and maintained for 8 h. The WCE was subjected to survivin ubiquitination analysis. (**I** and **J**) CNE2 cells expressing shCtrl or shSurvivin were transfected with Flag-survivin WT or T34A mutant, and cell viability was examined by MTS assay (**I**), and caspase 3 activity was examined by Caspase-3 Assay Kit (**J**). **p < 0.01, ***p < 0.001.

Phosphorylation of Thr34 stabilizes survivin^20^. We therefore determined whether Butein reduces survivin phosphorylation. The results showed that Butein suppressed survivin Thr34 phosphorylation dose-dependently in CNE2, HONE1, and SUNE1 cells (Fig. 4A). Treatment with Butein reduces Akt Ser473 phosphorylation and the downstream target Wee1 (Fig. 4B). Furthermore, down-regulation of Wee1 phosphorylation at Ser642 increased CDK1 phosphorylation on Tyr15. Consistently, the phosphorylation of CDK1 on Thr161, a marker for CDK1 activation, was suppressed dose-dependently (Fig. 4B). Knockdown of Akt with siRNA caused a reduction of the phosphorylation of Wee1 (Ser642), CDK1 (Thr161), and survivin (Thr34) (Fig. 4C). Overexpression of Myr-Akt1, a constitutively activated Akt, compromised Butein-induced decrease of phosphorylation of Wee1 (Ser642), CDK1 (Thr161), and survivin (Thr34) (Fig. 4D). The MTS data indicated that transfected with Myr-Akt1 rescued cell viability in Butein-treated CNE2 cells (Fig. 4E). Moreover, the activated cleaved-caspase 3 was down-regulated significantly (Fig. 4F), and cleaved-caspase 3 and -PARP expression was also inhibited (Fig. 4G). Mutation of survivin Thr34 to Ala34 caused stronger ubiquitination of survivin in Butein-treated CNE2 cells (Fig. 4H). Importantly, wild-type (WT) survivin, but not the T34A mutant, rescued cell viability (Fig. 4I) and reduced caspase 3 activity (Fig. 4J). Our data suggest that inhibition of Akt phosphorylation is required for survivin ubiquitination and degradation.

### Butein induces apoptosis in NPC cells

**Figure 5:**
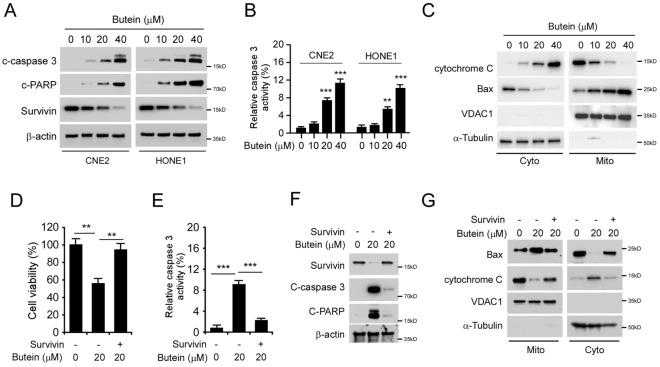
Butein promotes apoptosis. (**A** and **B**) CNE2 and HONE1 cells were treated with Butein for 24 h, the WCE was subjected to IB analysis (**A**), and caspase 3 activity was examined by Caspase 3 Assay Kit (**B**). **p < 0.01, ***p < 0.001. (**C**) CNE2 cells were treated with Butein for 24 h, and subcellular fractions were isolated and subjected to IB analysis. (**D**–**G**) CNE2 cells were transfected with survivin construct for 24 h, followed by Butein treated for another 24 h, cell viability was examined by MTS assay (**D**) and caspase 3 activity was examined by Caspase 3 Assay Kit (**E**). The WCE was subjected to IB analysis (**F**), and subcellular fractions were isolated and subjected to IB analysis (**G**). **p < 0.01, ***p < 0.001.

We further examined whether Butein activates apoptosis signaling in NPC cells. IB data showed that Butein increased the protein level of cleaved-PARP and -caspase 3 dose-dependently in CNE2 and HONE1 cells (Fig. 5A). Notably, caspase 3 was significantly elevated in Butein-treated cells (Fig. 5B), indicating that Butein promotes apoptosis in NPC cells. We next isolated the subcellular fractions in CNE2 cells after Butein treatment. The result showed that Butein promoted the release of cytochrome C from the mitochondrial to the cytoplasm. Consistently, the protein level of Bax in mitochondrial was upregulated along with the increased concentration of Butein (Fig. 5C). This evidence suggests that Butein activates apoptosis signaling. To determine whether Butein-induced apoptosis is related to survivin destruction, we overexpressed survivin in CNE2 cells. MTS data showed that overexpression of survivin restored cell viability (Fig. 5D), compromised apoptosis (Fig. 5E), and decreased the protein level of cleaved-caspase 3 and -PARP (Fig. 5F) in Butein-treated CNE2 cells. We further isolated subcellular fractions and found that overexpression of survivin rescued Butein promoted intrinsic apoptosis, as the release of cytochrome C from mitochondrial and the protein level of Bax in mitochondrial was decreased substantially (Fig. 5G). Our data suggest that Butein induces mitochondrial apoptosis in NPC cells.

### Butein inhibits in vivo tumor growth

**Figure 6:**
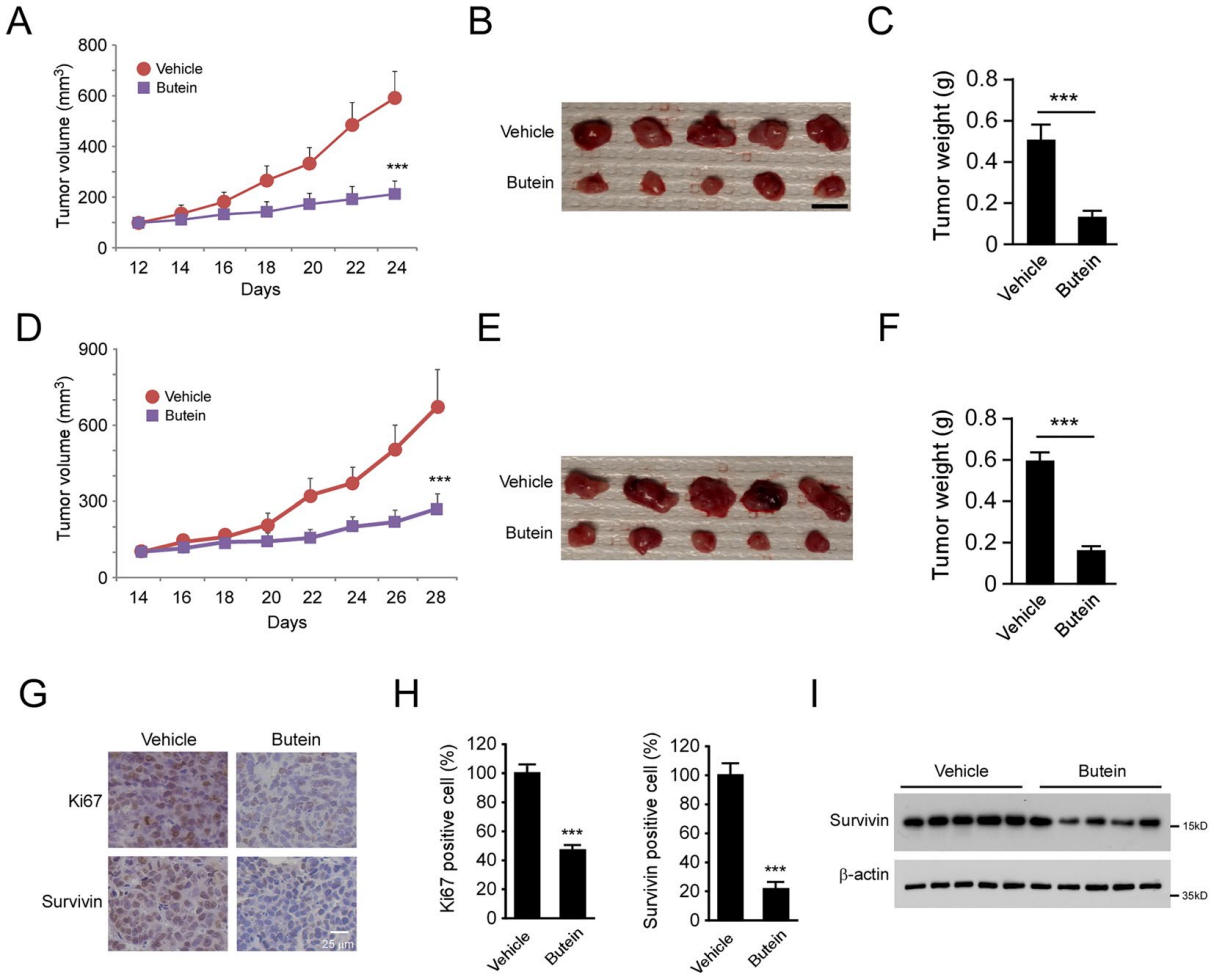
Butein suppresses the in vivo tumor growth. (**A**–**C**) The tumor volume (**A**), The image of tumor mass (**B**), and tumor weight (**C**) of CNE2-derived xenograft tumors treated with vehicle control or Butein. ***p < 0.001. (**D**–**F**) The tumor volume (**D**), The image of tumor mass (**E**), and tumor weight (**F**) of HONE1-derived xenograft tumors treated with Vehicle control or Butein. ***p < 0.001. (**G** and **H**) IHC staining (**G**) and quantitative analysis (**H**) of Ki67 and survivin expression in CNE2-derived xenograft tumors with vehicle or Butein treatment. ***p < 0.001. Scale bar, 25 μm. (**I**) IB analysis of survivin expression in CNE2-derived xenograft tumors with Vehicle or Butein treatment.

Xenograft mouse models were conducted using CNE2 and HONE1 cells to examine the in vivo anti-tumor effect of Butein. Our data showed that treatment with Butein significantly delayed CNE2 xenograft tumor growth, as two-thirds reduced the tumor volume compared to the vehicle-treated tumors (Fig. 6A). Moreover, the weight of tumor mass was down-regulated by over 60% in the Butein-treated group (Fig. 6B,C). Butein exhibited a similar inhibitory effect on HONE1 xenograft tumors (Fig. 6D–F). We further examined the protein level of survivin in CNE2-derived tumor tissues. The IHC results showed that Butein significantly decreased the population of Ki67-positive cells. The number of survivin-positive cells was reduced by nearly 80% in the Butein-treated group (Fig. 6G,H). IB data also revealed that the total protein level of survivin was reduced consistently (Fig. 6I). Our data indicate that Butein suppresses in vivo tumor development of CNE2 cells.

### Butein overcomes chemoresistance in NPC cells

**Figure 7:**
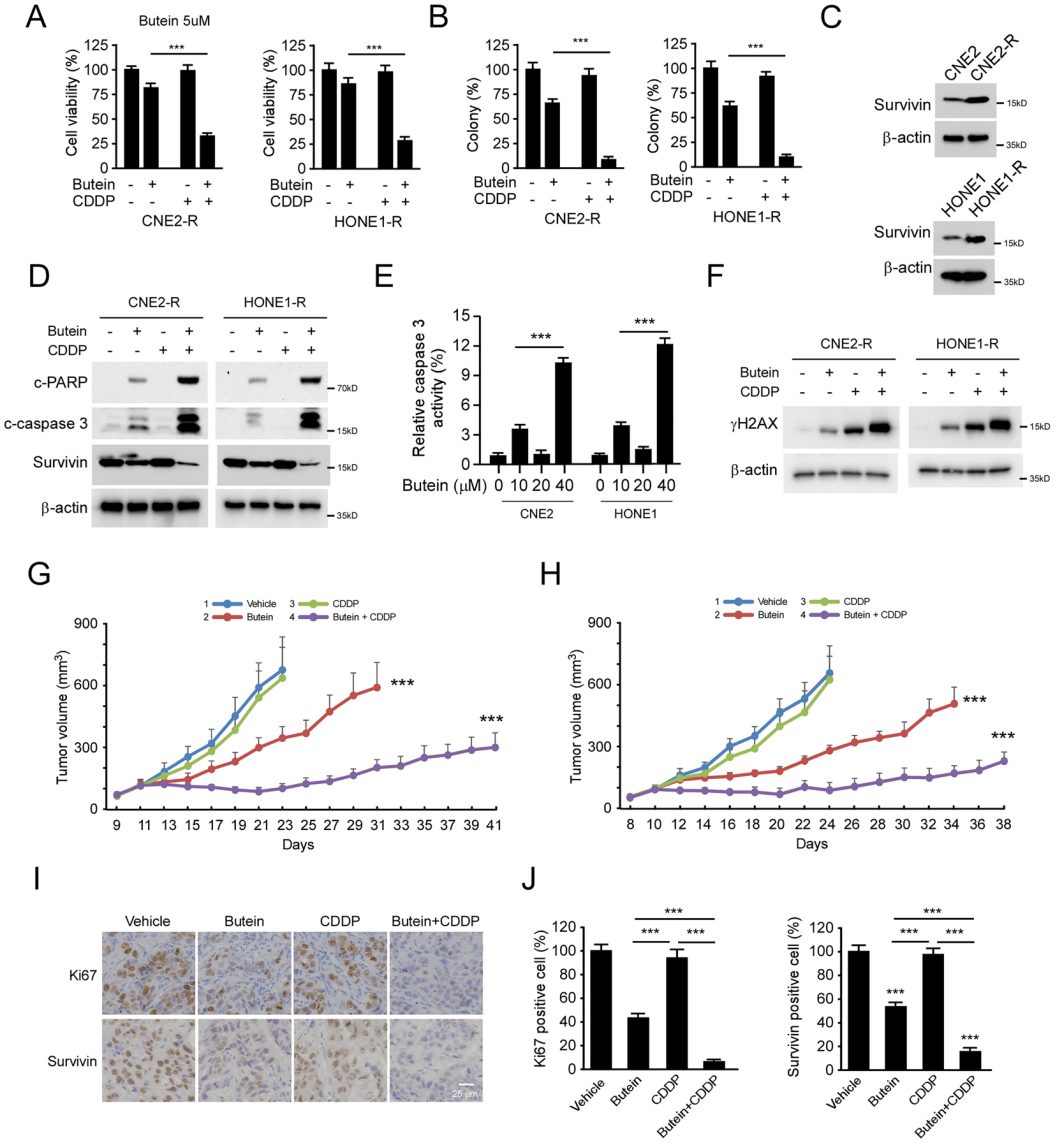
Butein overcomes CDDP resistance. (**A** and **B**) CDDP resistant CNE2-R (left) and HONE1-R (right) cells were treated with CDDP (3 µM), Butein (5 µM), or the combination for 24 h, cell viability and colony formation were examined by MTS (**A**) and soft agar (**B**) assays. (**C**) IB analysis of survivin expression in CNE2/ CNE2-IR and HONE1/ HONE1-R cells. (**D** and **E**) CNE2 and HONE1 cells were treated with CDDP (3 µM), Butein (5 µM), or the combination for 24 h, the WCE was subjected to IB analysis (**D**), and caspase 3 activity was examined by Caspase 3 Assay Kit (**E**). (**F**) CNE2 and HONE1 cells were treated with CDDP (3 µM), Butein (5 µM), or the combination for 24 h. The WCE was subjected to IB analysis. (**G** and **H**) In vivo tumor development of CNE2-R (**G**) and HONE1-R (**H**) cells treated with vehicle control, Butein, CDDP, or a Butein + CDDP combination. (**I** and **J**) IHC staining (**I**) and quantitative analysis (**J**) of Ki67 and survivin expression in HONE1-R-derived xenograft tumors with various treatments. ***p < 0.001.

To determine whether Butein affects chemotherapy of NPC cells, we first examined the inhibitory efficacy of Butein in combination with CDDP treatment in CNE2 and HONE1 cells. The MTS data showed that CDDP or Butein alone reduced cell viability by around 25–30%, whereas the combination treatment enhanced this anti-tumor efficacy by over 80% (Supplementary Fig. 3A). The colony formation was consistently blocked by over 90% in CNE2 and HONE1 cells after the combination treatment (Supplementary Fig. 3B). Importantly, these inhibitory effects were observed in CDDP-resistant CNE2-R and HONE1-R cells with Butein and CDDP treatment (Fig. 7A,B). The western blotting results revealed that survivin is overexpressed in chemoresistant CNE2-R and HONE1-R cells (Fig. 7C). Butein, but not CDDP alone, reduced the protein level of survivin and promoted the expression of cleaved-caspase 3/-PARP. In addition, caspase 3 activity increased with Butein treatment and further enhanced with the combination treatment (Fig. 7D,E), indicating that Butein induced apoptosis. The IB data showed that treatment with Butein facilitated CDDP-induced DNA damage, as the phosphorylation of H2AX was dramatically upregulated (Fig. 7F). We next examined whether Butein overcomes chemoresistance in vivo. The results showed that the CDDP alone failed to restrict the in vivo tumor development of CNE2-R and HONE1-R xenograft. However, Butein alone delayed tumor development, and the combination of Butein sensitized CNE2-R and HONE1-R xenograft to CDDP treatment (Fig. 7G,H), indicating that Butein overcomes chemoresistance of NPC cells in vivo. Moreover, the IHC staining showed that Butein, but not CDDP alone, reduced survivin protein levels in xenograft tumor tissues, and this inhibitory effect was further enhanced in the combination treatment group (Fig. 7I,J). These results indicate that Butein suppressed tumor growth and exhibited the potential to overcome CDDP resistance.

## Discussion

Butein (2′,3,4,4′-tetrahydroxychalcone) is a polyphenol compound found in several plants and has been found to exhibit potent antioxidant, anti-inflammatory, and also anti-tumor effects against multiple cancer types^21^. However, its effect on NPC and CDDP chemoresistance is not studied yet. Our data showed that Butein inhibited the cell viability of NPC cells and overcame chemoresistance by activating the intrinsic apoptosis signaling. Butein reduced the protein level of survivin dose-dependently and enhanced survivin ubiquitination by promoting the interaction between survivin and E3 ligase Fbxl7. Importantly, our data indicated that depletion of Fbxl7 compromised Butein-induced survivin destruction. Our data discovered a novel anti-tumor mechanism of Butein and suggested that targeting survivin is a promising alternative strategy for NPC treatment.

Survivin is frequently dysregulated in human cancers. The high protein level of survivin accelerates cell cycle progression and confers chemo/radioresistance by suppressing the apoptosis cascade. For instance, survivin highly expressed head and neck squamous carcinoma cells (HNSCC) exhibited a robust therapeutic resistance towards lapatinib^22^. Downregulation of survivin overcomes osimertinib and radiotherapy resistance in glioma cells and oral squamous cell carcinoma cells^23,24^, respectively. Recent studies indicate that overexpression of survivin enhanced angiogenesis and metastasis. Pharmaceutical inhibition of survivin, or reduction of survivin protein level, suppressed metastasis of breast^25^, ovarian^26^, and lung cancer^27^. Moreover, the high protein level of survivin is corrected with the poor prognosis of HNSCC and NPC^28–30^. Our data revealed that survivin depletion blunted the malignant phenotype of NPC cells, including the inhibitory effects on cell viability, colony formation, and in vivo tumor development. This evidence suggests that targeting survivin is an alternative strategy for clinical treatment in multiple cancer models.

The expression of survivin is tightly regulated by ubiquitination in mammalian cells. Dysfunction of ubiquitination-mediated degradation is considered a major mechanism to cause the accumulation of survivin in human cancer cells. So far, two E3 ligases, the F-box Protein Fbxl7 and the X-linked inhibitor of apoptosis (XIAP) have been identified to catalyze survivin ubiquitination^31,32^. In addition, overexpression of deubiquitinase, such as CSN5 and FAT10^33,34^, can specifically remove the K-48 linked ubiquitination chains, stabilize survivin, and inhibit apoptosis in humans cancer cells. Recent studies showed that targeting survivin induced cancer cell apoptosis and enhanced radio/chemotherapy-mediated tumor-killing efficacy. Silencing survivin using antibody-conjugated poly (Propylene Imine)-based polyplexes inhibits the growth of PSCA-positive tumors^35^. MiR-34a-mediated survivin inhibition improves the antitumor activity of selinexor in triple-negative breast cancer^36^. Translational suppression of survivin expression by natural product licochalcone A inhibits non-small cell lung cancer cells^37^. Moreover, reduced expression of BIRC5 by Salinomycin and xanthohumol enhances radiotherapy sensitivity of NPC and oral squamous cell carcinoma cells, respectively^23,38^. In the present study, we found that Butein promoted the interaction between survivin and Fbxl7, facilitating Fbxl7-mediated ubiquitination in NPC cells. We discovered that Butein suppressed survivin Thr34 phosphorylation by inhibiting Akt-Weel-CDK1 signaling, and overexpression of activated Akt1 rescued Butein-induced survivin destruction. YM155^39,40^ is a survivin suppressant that can specifically inhibit the transcription of survivin by disrupting the binding of Sp1 and the core promoter of survivin, exerting a robust anti-tumor activity in human cancer cells. Our results indicate that Butein is a novel survivin inhibitor that exhibits the anti-tumor effect by decreasing the stability of survivin protein.

Overall, this study suggests that a high protein level of survivin is required for maintaining the malignant phenotype of NPC cells. The natural compound Butein inhibits NPC cells by downregulation of survivin in an Akt-Weel-CDK1 signaling-dependent manner. We demonstrated that Butein inhibits survivin phosphorylation, which induced Fbxl7-induced survivin ubiquitination and degradation. Our study extends the anti-tumor mechanism of Butein and suggests that Butein is a promising therapeutic agent for NPC treatment.

## Supplementary Information

Supplementary Information.

## Data Availability

Materials are available upon request.

## References

[1] Yarza R, Bover M, Agullo-Ortuno MT, Iglesias-Docampo LC (2021). Current approach and novel perspectives in nasopharyngeal carcinoma: The role of targeting proteasome dysregulation as a molecular landmark in nasopharyngeal cancer. J. Exp. Clin. Cancer Res..

[2] Chang ET, Ye W, Zeng YX, Adami HO (2021). The evolving epidemiology of nasopharyngeal carcinoma. Cancer Epidemiol. Biomark. Prev..

[3] Looi CK (2021). Roles of inflammasomes in Epstein–Barr virus-associated nasopharyngeal cancer. Cancers.

[4] Liu W (2021). The diagnostic value of EBV-DNA and EBV-related antibodies detection for nasopharyngeal carcinoma: A meta-analysis. Cancer Cell Int..

[5] Su L, She L, Shen L (2020). The current role of adjuvant chemotherapy in locally advanced nasopharyngeal carcinoma. Front. Oncol..

[6] Chen YP (2021). Chemotherapy in combination with radiotherapy for definitive-intent treatment of stage II-IVA nasopharyngeal carcinoma: CSCO and ASCO guideline. J. Clin. Oncol..

[7] Guan S, Wei J, Huang L, Wu L (2020). Chemotherapy and chemo-resistance in nasopharyngeal carcinoma. Eur. J. Med. Chem..

[8] Altieri DC (2008). Survivin, cancer networks and pathway-directed drug discovery. Nat. Rev. Cancer.

[9] Warrier NM, Agarwal P, Kumar P (2020). Emerging importance of survivin in stem cells and cancer: The development of new cancer therapeutics. Stem Cell Rev. Rep..

[10] Dai CH (1864). YM155 sensitizes non-small cell lung cancer cells to EGFR-tyrosine kinase inhibitors through the mechanism of autophagy induction. Biochim. Biophys. Acta Mol. Basis Dis..

[11] Wang T, Chen Y, Goodale D, Allan AL, Ronald JA (2021). A survivin-driven, tumor-activatable minicircle system for prostate cancer theranostics. Mol. Ther. Oncolytics.

[12] Touloumis Z, Lazaris A, Griniatsos J (2020). The prognostic significance of Caspase-3 and survivin expression in colorectal cancer patients. J. BUON.

[13] Pakbin B (2021). Probiotic *Saccharomyces cerevisiae* var. boulardii supernatant inhibits survivin gene expression and induces apoptosis in human gastric cancer cells. Food Sci. Nutr..

[14] Kapiris I (2019). Survivin expression in hepatocellular carcinoma. Correlation with clinicopathological characteristics and overall survival. J. BUON.

[15] Frazzi R (2021). BIRC3 and BIRC5: Multi-faceted inhibitors in cancer. Cell Biosci..

[16] Huang YH, Yeh CT (2019). Functional compartmentalization of HSP60-survivin interaction between mitochondria and cytosol in cancer cells. Cells.

[17] Rafatmanesh A (2020). The survivin molecule as a double-edged sword in cellular physiologic and pathologic conditions and its role as a potential biomarker and therapeutic target in cancer. J. Cell Physiol..

[18] Li M (2021). Targeting Aurora B kinase with Tanshinone IIA suppresses tumor growth and overcomes radioresistance. Cell Death Dis..

[19] Yu X (2019). Skp2-mediated ubiquitination and mitochondrial localization of Akt drive tumor growth and chemoresistance to cisplatin. Oncogene.

[20] O'Connor DS (2000). Regulation of apoptosis at cell division by p34cdc2 phosphorylation of survivin. Proc. Natl. Acad. Sci. USA.

[21] Tuli HS (2021). Molecular mechanisms underlying chemopreventive potential of butein: Current trends and future perspectives. Chem. Biol. Interact..

[22] Lehman CE (2019). Survivin in insulin-like growth factor-induced resistance to lapatinib in head and neck squamous carcinoma cells. Front. Oncol..

[23] Li M (2020). Promotion of ubiquitination-dependent survivin destruction contributes to xanthohumol-mediated tumor suppression and overcomes radioresistance in human oral squamous cell carcinoma. J. Exp. Clin. Cancer Res..

[24] Suzuki S (2019). Brexpiprazole, a serotonin-dopamine activity modulator, can sensitize glioma stem cells to osimertinib, a third-generation EGFR-TKI, via survivin reduction. Cancers.

[25] Oparina N (2021). Prognostic significance of BIRC5/survivin in breast cancer: Results from three independent cohorts. Cancers.

[26] Zhao G (2019). Ovarian primary and metastatic tumors suppressed by survivin knockout or a novel survivin inhibitor. Mol. Cancer Ther..

[27] Tang Q (2021). Mutant p53 regulates survivin to foster lung metastasis. Genes Dev..

[28] Xie W, Yan O, Liu F, Han Y, Wang H (2021). Prognostic value of survivin in nasopharyngeal carcinoma: A systematic review and meta-analysis. J. Cancer.

[29] Zhou LQ, Hu Y, Xiao HJ (2021). The prognostic significance of survivin expression in patients with HNSCC: A systematic review and meta-analysis. BMC Cancer.

[30] Jin PY (2019). Roles of beta-catenin, TCF-4, and survivin in nasopharyngeal carcinoma: Correlation with clinicopathological features and prognostic significance. Cancer Cell Int..

[31] Arora V (2007). Degradation of survivin by the X-linked inhibitor of apoptosis (XIAP)-XAF1 complex. J. Biol. Chem..

[32] Liu Y (2015). The proapoptotic F-box protein Fbxl7 regulates mitochondrial function by mediating the ubiquitylation and proteasomal degradation of survivin. J. Biol. Chem..

[33] Li J, Li Y, Wang B, Ma Y, Chen P (2018). CSN5/Jab1 facilitates non-small cell lung cancer cell growth through stabilizing survivin. Biochem. Biophys. Res. Commun..

[34] Dong D (2016). Ubiquitin-like protein FAT10 promotes bladder cancer progression by stabilizing survivin. Oncotarget.

[35] Jugel W (2021). Targeted RNAi of BIRC5/survivin using antibody-conjugated poly(propylene imine)-based polyplexes inhibits growth of PSCA-positive tumors. Pharmaceutics.

[36] Martini S (2021). miR-34a-mediated survivin inhibition improves the antitumor activity of selinexor in triple-negative breast cancer. Pharmaceuticals.

[37] Gao F (2021). Licochalcone A inhibits EGFR signalling and translationally suppresses survivin expression in human cancer cells. J. Cell Mol. Med..

[38] Chang Y, Geng Q, Bao Q, Hu P (2020). Salinomycin enhances radiotherapy sensitivity and reduces expressions of BIRC5 and NEIL2 in nasopharyngeal carcinoma. Eur. Rev. Med. Pharmacol. Sci..

[39] Tolcher AW (2008). Phase I and pharmacokinetic study of YM155, a small-molecule inhibitor of survivin. J. Clin. Oncol..

[40] Ryan BM, O'Donovan N, Duffy MJ (2009). Survivin: A new target for anti-cancer therapy. Cancer Treat. Rev..

