# Author Correction: Butein promotes ubiquitination-mediated survivin degradation inhibits tumor growth and overcomes chemoresistance

**DOI:** 10.1038/s41598-023-28989-z

**Published:** 2023-01-31

**Authors:** Xin Dong, Wenbin Liu, Xiaoying Li, Yu Gan, Li Zhou, Wei Li, Li Xie

**Affiliations:** 1 Department of Head and Neck Surgery, Hunan Cancer Hospital/The Affiliated Cancer Hospital of Xiangya School of Medicine, Central South University, Changsha, 410013 Hunan China; 2 Department of Pathology, Hunan Cancer Hospital/The Affiliated Cancer Hospital of Xiangya School of Medicine, Central South University, Changsha, 410013 Hunan China; 3 Department of Pathology, National Clinical Research Center for Geriatric Disorders, Xiangya Hospital of Central South University, Changsha, 410008 Hunan China; 4 Cell Transplantation and Gene Therapy Institute, The Third Xiangya Hospital, Central South University, Changsha, 410013 Hunan China; 5 Department of Clinical Laboratory, National Cancer Center/National Clinical Research Center for Cancer/Cancer Hospital, Chinese Academy of Medical Sciences and Peking Union Medical College, Beijing, 100021 China

Correction to: *Scientific Reports* 10.1038/s41598-022-21839-4, published online 30 November 2022

In the original version of this Article, Xin Dong was incorrectly affiliated with ‘Department of Head and Neck Surgery, Hunan Cancer Hospital/The Affiliated Cancer Hospital of Xiangya School of Medicine, Central South University, Changsha 410013, Hunan, China’.

The correct affiliation is listed below.

Department of Clinical Laboratory, National Cancer Center/National Clinical Research Center for Cancer/Cancer Hospital, Chinese Academy of Medical Sciences and Peking Union Medical College, Beijing 100021, China.

The original Article has been corrected.

