# Supplementary material for: Butein promotes ubiquitination-mediated survivin degradation inhibits tumor growth and overcomes chemoresistance
Source: Sci Rep. 2022 Nov 30;12:20644. doi: 10.1038/s41598-022-21839-4 (PMC9712619; doi:10.1038/s41598-022-21839-4)

Supplementary Figure Legends

Supplementary Figure 1. The top10 candidates exhibited a more potent inhibitory effect on CNE2 cells from natural compound screening.

Supplementary Figure 2. Butein promotes survivin ubiquitination. A, HONE1 cells were treated with Butein for 24 h, and incubated with MG132 (20 μM) for 8 h. The WCE was subjected to survivin ubiquitination analysis. B, HONE1 cells were transfected with various constructs for 24 h, followed by Butein treated for 24 h, and incubated with MG132 (20 μM) for 8 h. The WCE was subjected to survivin ubiquitination analysis. C, HONE1 cells were transfected with siFbxl7 for 24 h and treated with Butein for another 24 h. WCE was collected after the cells were incubated with MG132 (20 μM) for 8 h. The WCE was subjected to survivin ubiquitination analysis. D, HONE1 cells were transfected with various constructs for 24 h, followed by Butein treated for 24 h, and incubated with MG132 (20 μM) for 8 h. The WCE was subjected to survivin ubiquitination analysis. E, HONE1 cells were transfected with various constructs for 24 h, followed by Butein treated for 24 h, and incubated with MG132 (20 μM) for 8 h. The WCE was subjected to survivin ubiquitination analysis.

Supplementary Figure 3. A and B, CNE2 (left) and HONE1 (right) cells were treated with CDDP (3 μM), Butein (5 μM), or the combination for 24 h, cell viability and colony formation were examined by MTS (A) and soft agar (B) assays. ***, $p<0.001$.

Supplementary Figure 4. The full image of the immunoblotting result.

Supplementary Figure 1

Compounds	Cell viability(%)
Synephrine	82.7
Quercetin	82.7
Myricitrin	82.3
Caffeic Acid	80.7
Laetrile	80.3
(-)-Epogallocatechin Gallate	77
Isoliquiritigenin	76.7
Fisetin	76
Cytisine	74
Butein	69.2

Supplementary Figure 2

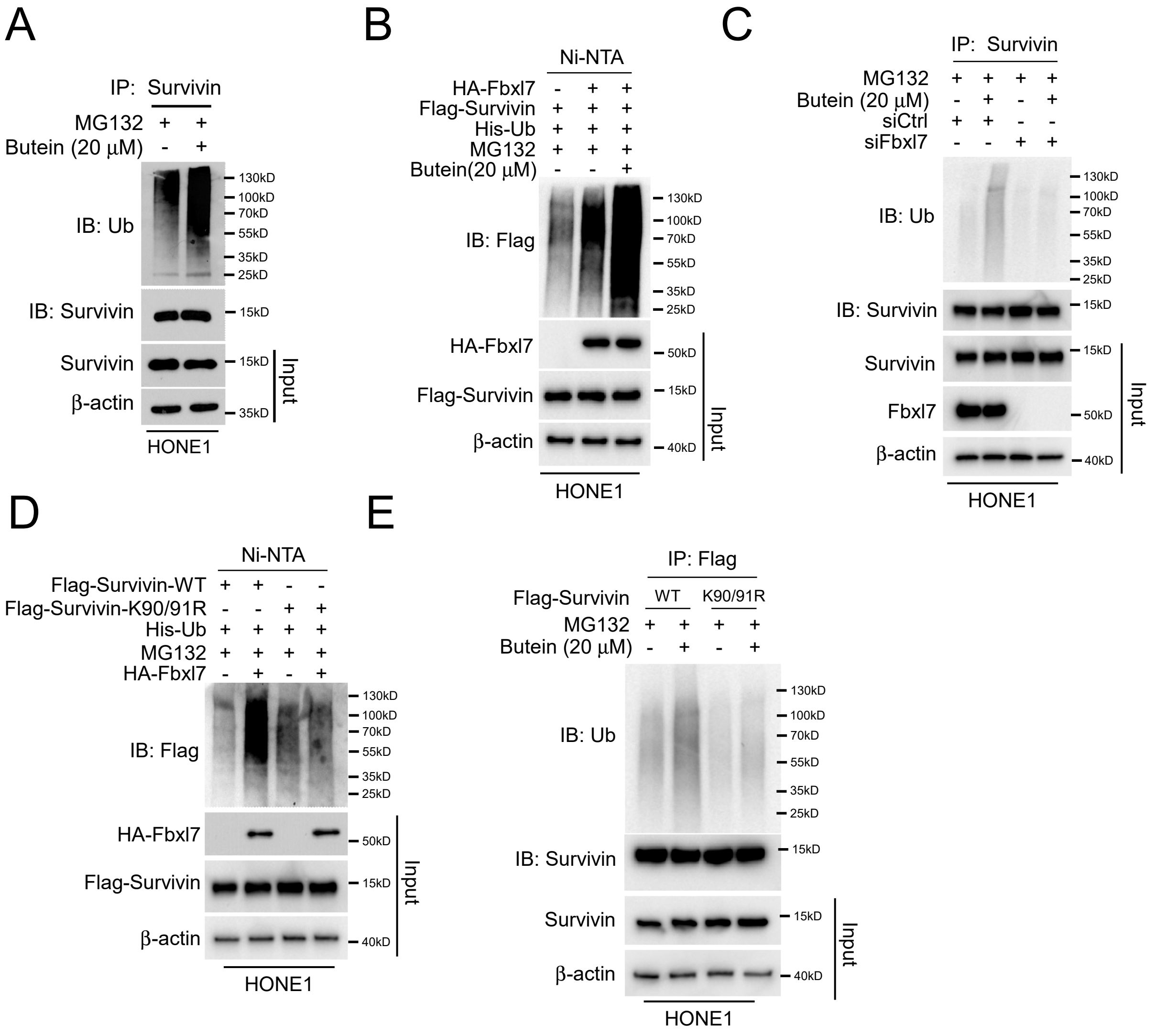

Supplementary Figure 3

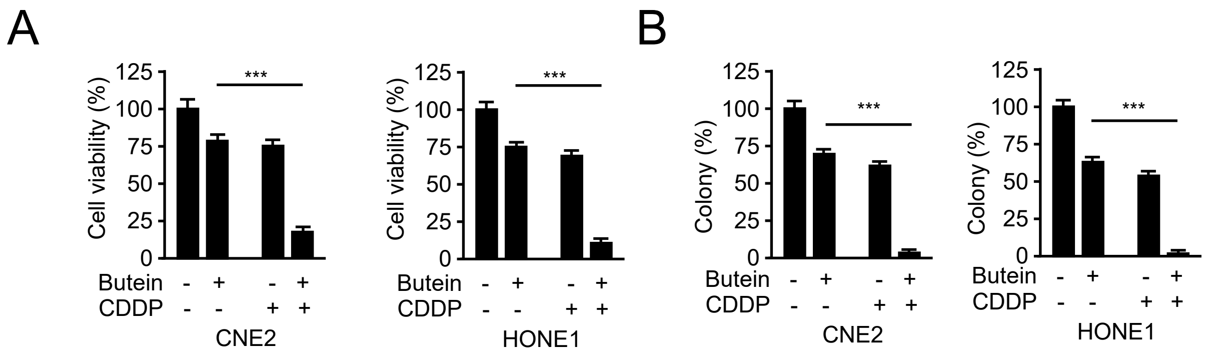

Supplementary Table 1

Compounds	
Apigenin	Quercetin Dihydrate
Cytisine	Silymarin
Icariin	Troxerutin
Phloretin	Rutaecarpine
Sclareol	Oxymatrine
Tangeretin	Sinomenine
(-)-Epigallocatechin Gallate	Naringin
Arbutin	Luteolin
Bilobalide	Honokiol
Gossypol	Indirubin
Nobiletin	Cryptotanshinone
Sclareolide	Baicalin
Piperine	Laetrile
Taxifolin (Dihydroquercetin)	Andrographolide
Artesunate	Cyclosporin A
Caffeic Acid	Bergenin
Gramine	Magnolol
Limonin	Paeonol
Osthole	Rutin
Neohesperidin	Sesamin
Salicin	Curcumol
Myricetin	Xanthone
Oridonin	Gastrodin
Kinetin	Synephrine
Tanshinone I	Sorbitol
Puerarin	Rotundine
Silibinin	Hematoxylin
Enoxolone	geniposidicacid
Hesperetin	aloe-emodin
Myricitrin	progesterone
Azomycin	dihydroquercetian
Chrysin	polydatin
Esculin	Sinomenine
Baicalein	aloin
Butein	hordenine
Emodin	phloretin
Cinchonidine	Quercetin
Fisetin	Isoliquiritigenin
Hesperidin	Indirubin
Kaempferol	genipin
Phlorizin	tretinoin

Supplementary Figure 4

Supplementary Figure 4a. Original image of Figure 1

C

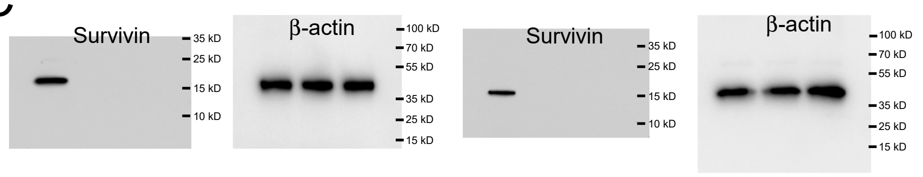

Supplementary Figure 4b. Original image of Figure 3

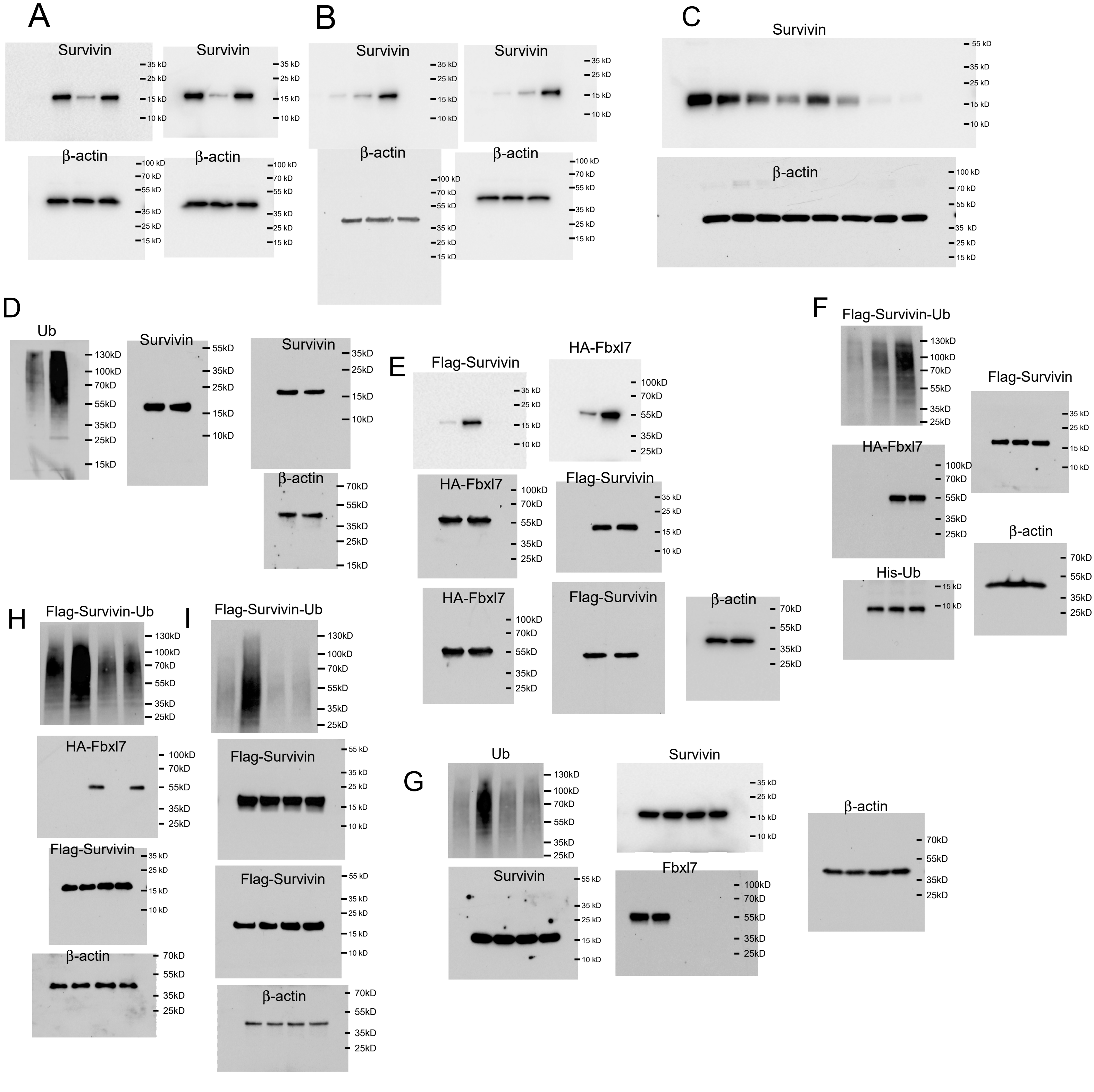

Supplementary Figure 4c. Original image of Figure 4

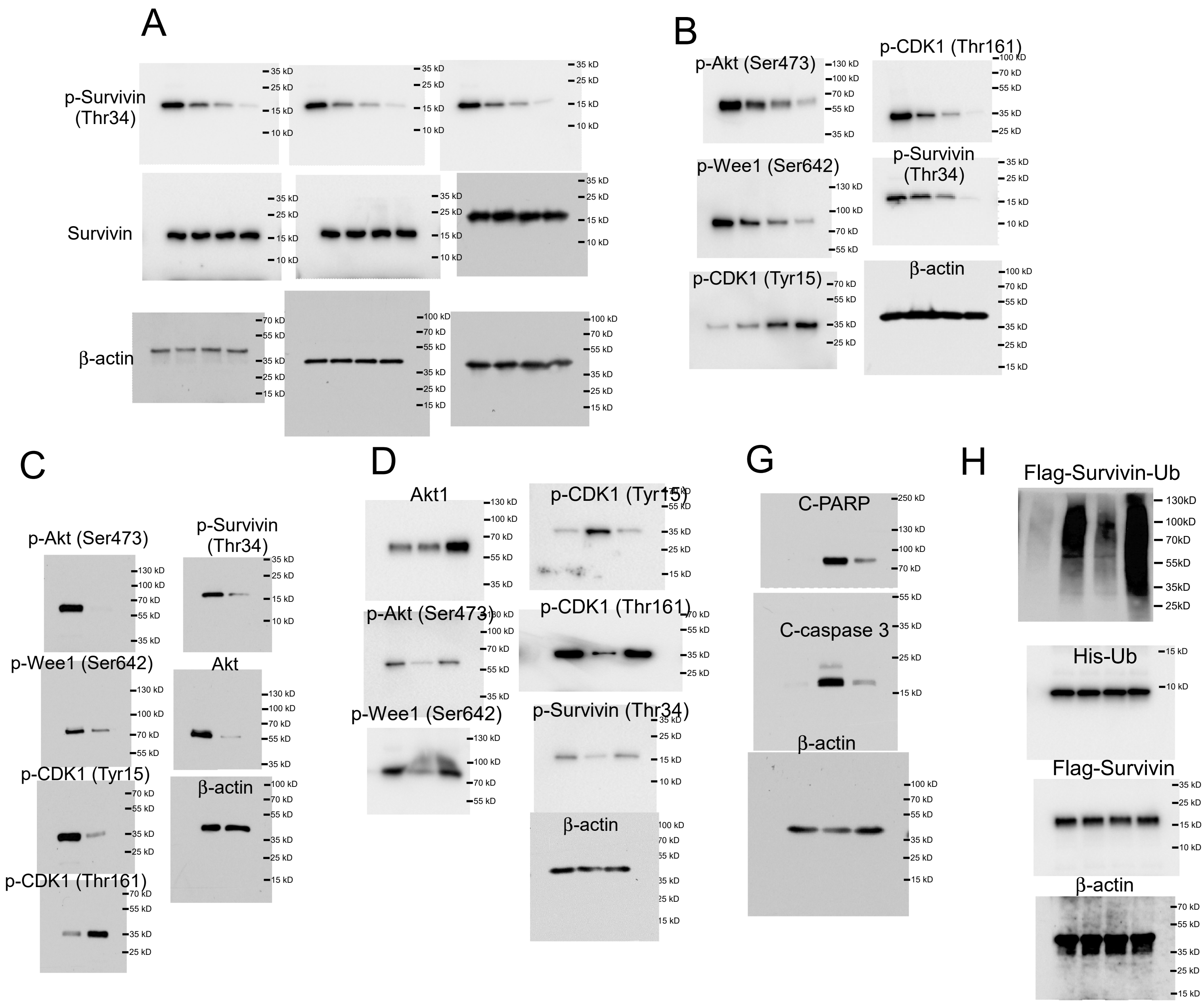

Supplementary Figure 4d. Original image of Figure 5

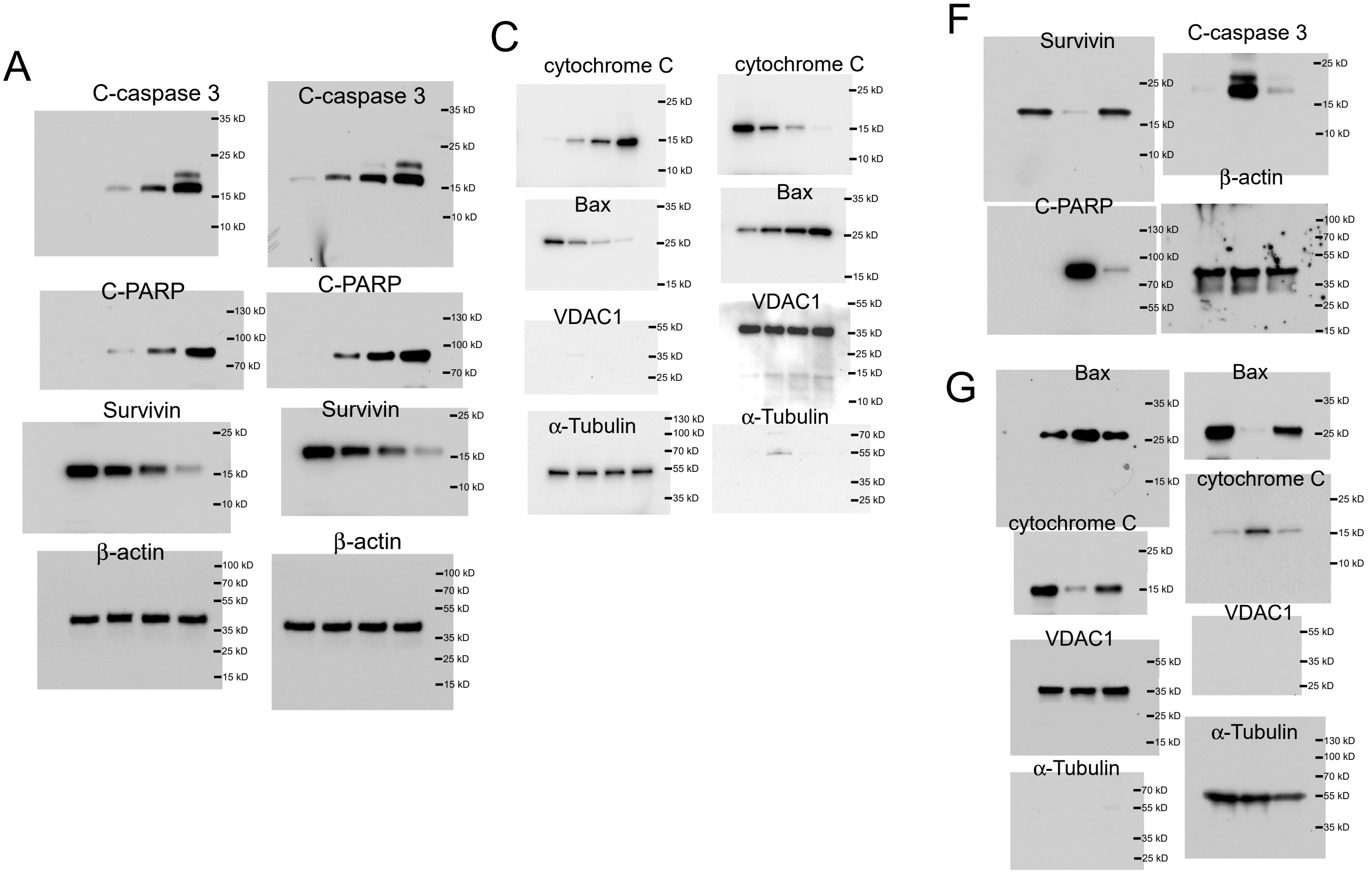

Supplementary Figure 4e. Original image of Figure 6

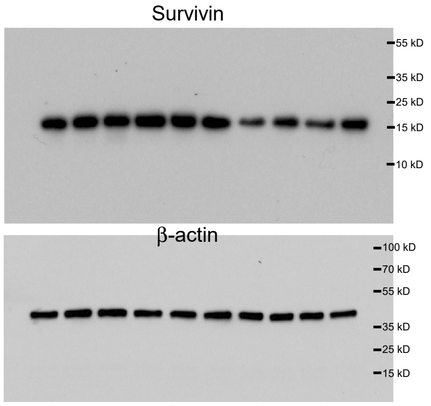

Supplementary Figure 4f. Original image of Figure 7

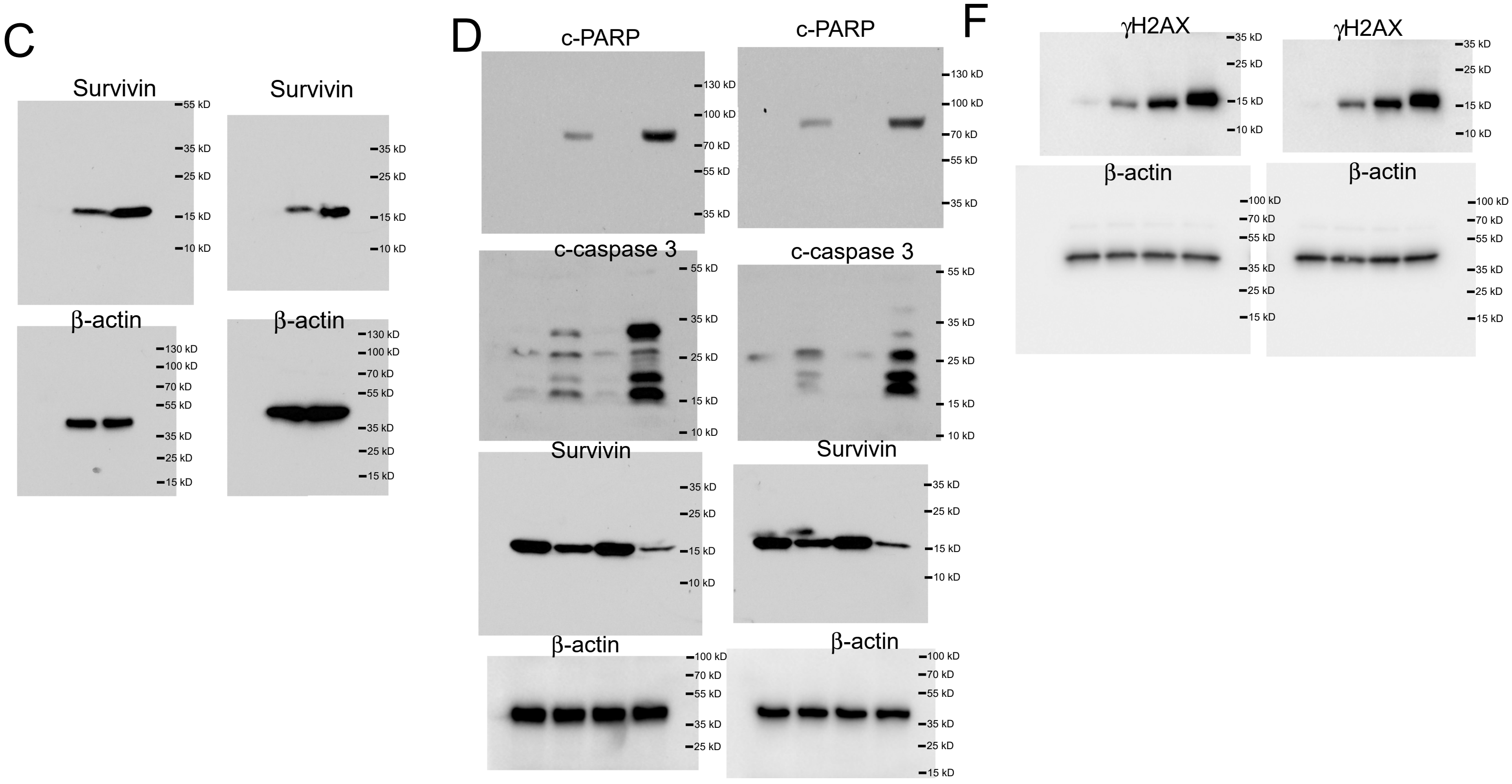

Supplementary Figure 4g. Original image of Supplementary Figure 2

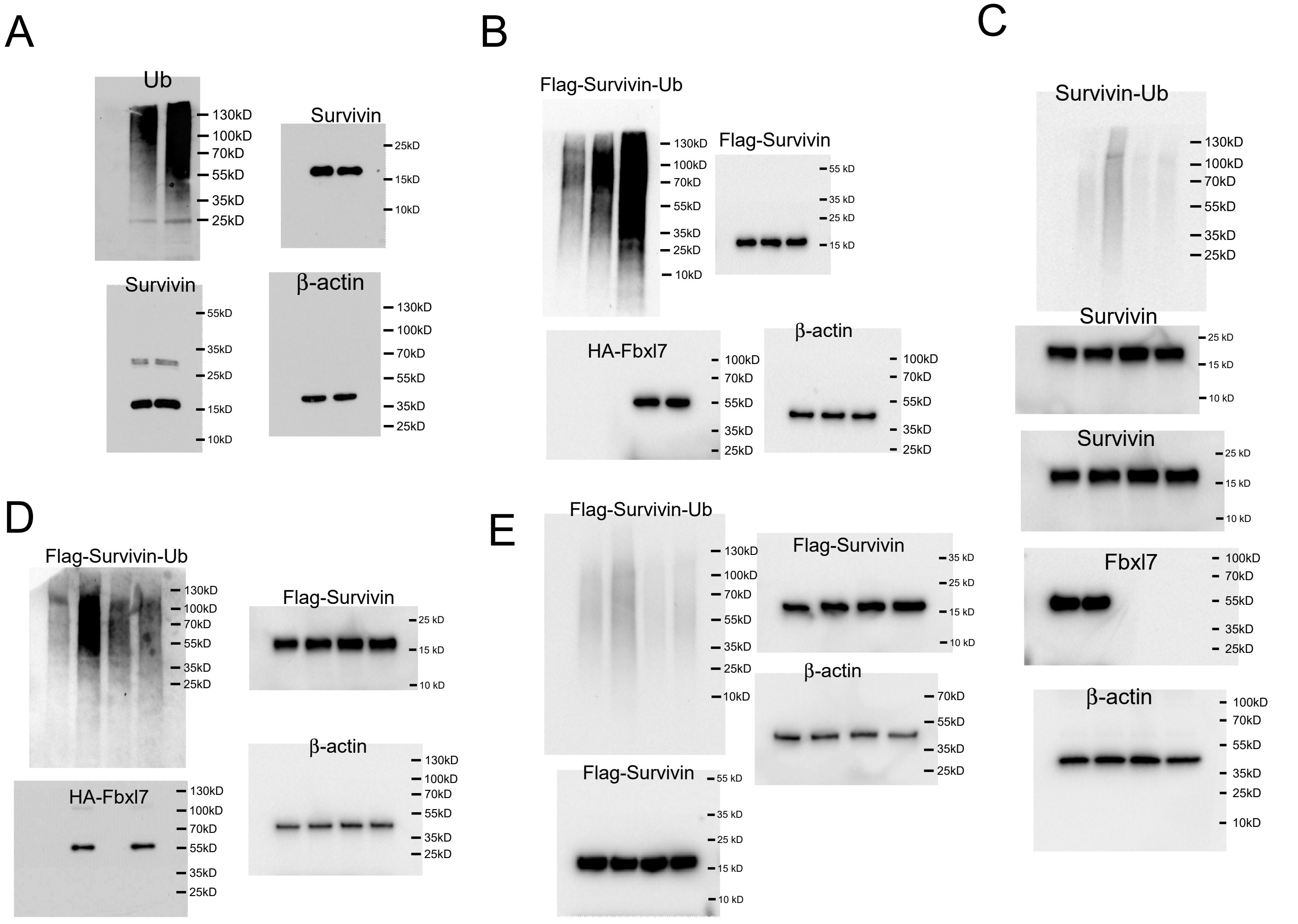

Supplement: Supplementary file 1 — Supplementary Information. [file 41598_2022_21839_MOESM1_ESM.pdf]
